# Arbuscular Mycorrhizal Fungi Induce Changes of Photosynthesis-Related Parameters in Virus Infected Grapevine

**DOI:** 10.3390/plants12091783

**Published:** 2023-04-26

**Authors:** Emanuel Gaši, Tomislav Radić, Mate Čarija, Giorgio Gambino, Raffaella Balestrini, Katarina Hančević

**Affiliations:** 1 Institute for Adriatic Crops and Karst Reclamation, Put Duilova 11, 21000 Split, Croatia; emanuel.gasi@krs.hr (E.G.); mate.carija@krs.hr (M.Č.); katarina.hancevic@krs.hr (K.H.); 2 Institute for Sustainable Plant Protection, National Research Council (IPSP-CNR), Strada delle Cacce 73, 10135 Torino, Italy; giorgio.gambino@ipsp.cnr.it (G.G.); raffaella.balestrini@ipsp.cnr.it (R.B.)

**Keywords:** GRSPaV, *Rhizophagus irregularis*, *Funneliformis mosseae*, *Funneliformis caledonium*, GLRaV-3, GPGV, net photosynthesis rate, chlorophyll

## Abstract

The negative effects of viruses and the positive effects of arbuscular mycorrhizal fungi (AMF) on grapevine performance are well reported, in contrast to the knowledge about their interactive effects in perennial plants, e.g., in grapevine. To elucidate the physiological consequences of grapevine–AMF–virus interactions, two different AMF inoculum (*Rhizophagus irregularis* and ‘Mix AMF’) were used on grapevine infected with grapevine rupestris stem pitting virus, grapevine leafroll associated virus 3 and/or grapevine pinot gris virus. Net photosynthesis rate (A_N_), leaf transpiration (E), intercellular CO_2_ concentration (C_i_) and conductance to H_2_O (g_s_) were measured at three time points during one growing season. Furthermore, quantum efficiency in light (Φ_PSII_) and electron transport rate (ETR) were surveyed in leaves of different maturity, old (basal), mature (middle) and young (apical) leaf. Lastly, pigment concentration and growth parameters were analysed. Virus induced changes in grapevine were minimal in this early infection stage. However, the AMF induced changes of grapevine facing biotic stress were most evident in higher net photosynthesis rate, conductance to H_2_O, chlorophyll a concentration, total carotenoid concentration and dry matter content. The AMF presence in the grapevine roots seem to prevail over virus infection, with *Rhizophagus irregularis* inducing greater photosynthesis changes in solitary form rather than mixture. This study shows that AMF can be beneficial for grapevine facing viral infection, in the context of functional physiology.

## 1. Introduction

Grapevine (*Vitis vinifera* L.) is one of the most important perennial crops globally, with viral pathogens posing a great threat to the viticulture through major economic loses [1]. With more than 80 viral species associated with the grapevine host, it represents one of the most virus-prone crops [2]. The effects of viral diseases on grapevine is a complex research topic, including changes in primary and secondary metabolites, photosynthesis, oxidative stress, antioxidative metabolism and cellular alterations [3,4,5,6]. Therefore, grapevine photosynthesis remains the point of interest for virus-induced damage across different virus–grapevine cultivar systems [7]. The severity of photosynthetic perturbations in the grapevine is dependent on the viral species, with pernicious grapevine viruses (e.g., grapevine leafroll-associated virus 3, GLRaV-3) accounting for more detrimental consequences on photosynthesis [8,9,10,11,12]. Some new emerging viruses, such as grapevine pinot gris virus (GPGV) may cause severe consequences, but its influence on the host physiology, such as photosynthesis, is underexplored [13]. However, many grapevine viruses are latent without triggering apparent phenotypic changes and with underexplored influence on grapevine physiology [1]. Grapevine rupestris stem-pitting associated virus (GRSPaV) is considered ubiquitous with seemingly asymptomatic infection [14], but recent works point to possible beneficial role of this virus on grapevine [15,16]. The presence of GRSPaV positively influences grapevine growth regardless of lower net photosynthetic rate and CO_2_ assimilation induced by virus infection [17]. Therefore, GRSPaV, despite being one of the most widely spread, represents a virus with unique and still unclear pathology.

The grapevine, however, tends to form mutualistic relationship with arbuscular mycorrhizal fungi (AMF) in the rhizosphere [18,19]. The mycorrhizal association greatly contributes to grapevine growth and nutrition [20,21]. Moreover, positive impact of AMF has been reported in grapevine exposed to numerous abiotic stresses through improvement of leaf water status, photosynthetic activity and chlorophyll concentration [22]. In addition, the remedial properties of AMF are described in grapevine facing biotic stresses. [23]. So far, the induction of defense response has been shown in grapevine inoculated by *Rhizophagus irregularis* and with subsequent infection by *Plasmopara viticola* or *Botrytis cinerea* [24]. The authors observed changes in stilbenoid biosynthesis pathways and argue that mycorrhizal fungi could enhance defense response against aerial pathogens [24]. Similarly, bioprotective effects of AMF has been shown against grapevine attacking ectoparasitic nematode *Xiphinema index*, where local and systemic defense processes were activated in the grapevine as a consequence of previously established mycorrhizal symbiosis [25]. The indirect mycorrhizal protection against the grapevine fanleaf virus (GFLV), born by aforementioned nematode species, has been shown in the mycorrhizal grapevine through inhibition of nematode transmission [26]. For investigating AMF alleviation of biotic stress induced by virus infection most works have been done on herbaceous crops, while studies on perennial plants, e.g., grapevine are fairly obscure. Nevertheless, a significant progress has been made in unraveling this complex interaction. So far, there have been reports of mycorrhiza induced resistance (MIR) based on induction of plant defense pathways [27], but also mycorrhiza induced susceptibility (MIS) defined through higher viral replication and intensified symptom development [28]. Few comprehensive reviews have systematically summarized research involving different plant hosts, AMF and virus species [28,29,30]. Recent studies showed AMF stimulated priming effects in virus infected tomato plants through mitigating physiological discrepancies and symptom development caused by viral infection [31]. Further, interesting study using same AMF species and host plant, but different viral species showed differential response regarding viral accumulation [32]. Therefore, the host response to the viral infection is not simply dependent on the relationship with AMF, rather the properties of each individual partner, e.g., lifestyle, species and genotype [29,33].

The physiological processes of the grapevine, in the light of multiple interactions regarding viral pathogens and symbiotic fungi (e.g., arbuscular mycorrhizal fungi), are vastly under-investigated, despite being predominantly present in agroecosystems in vineyards worldwide. Since grapevine is increasingly gaining status of a model organism for all fruit trees species, it serves as a perfect candidate for investigating above described complex interactions influencing plant physiology [1]. Therefore, the aim of this paper is to explore the physiological changes in the grapevine induced by arbuscular mycorrhizal fungi in the light of different severities of viral biotic stress. For that purpose, the GRSPaV will be used as a less pathogenic stress inducer, and GRSPaV coinfection with GLRaV-3 and/or GPGV will be used as a source of stronger pathogenic stress induction in the grapevine. The grapevine photosynthetic physiology processes and growth parameters will be the main interest in evaluating the effects of this multi-interactive biosystem.

## 2. Results

### 2.1. Root Colonization with AMF

Prior to AMF inoculation, grapevine plants were subjected to detection of virus presence and virus combinations used are presented in the Table 1. Inoculation of selected grapevine treatments with AMF resulted in high total root colonization and also in high arbuscules and variable vesicles colonization as shown in Table 1. High level of total AMF colonization was a prerequisite for evaluating AMF influence on grapevine photosynthesis, which was the aim of the study.

Total arbuscular mycorrhizal colonization and colonization by arbuscules, vesicles and hyphae did not depend on virus inoculum, but varied with type of AMF inoculum to a statistically significant level, as expected. This was confirmed by two-way ANOVA which gave no virus × AMF interaction (*p* < 0.05, Table 1) but strong dependence on AMF status. Application of two different AMF inoculums resulted in significantly higher colonization of arbuscules and vesicles and of total AMF colonization in treatments with only *Rhizophagus irregularis*, compared to treatments inoculated with mix of AMF species. Presence of microscopic intersections with hyphae only showed the opposite pattern, being more abundant in treatments with mix AMF species applied.

### 2.2. Photosynthesis Analysis

**Figure 1:**
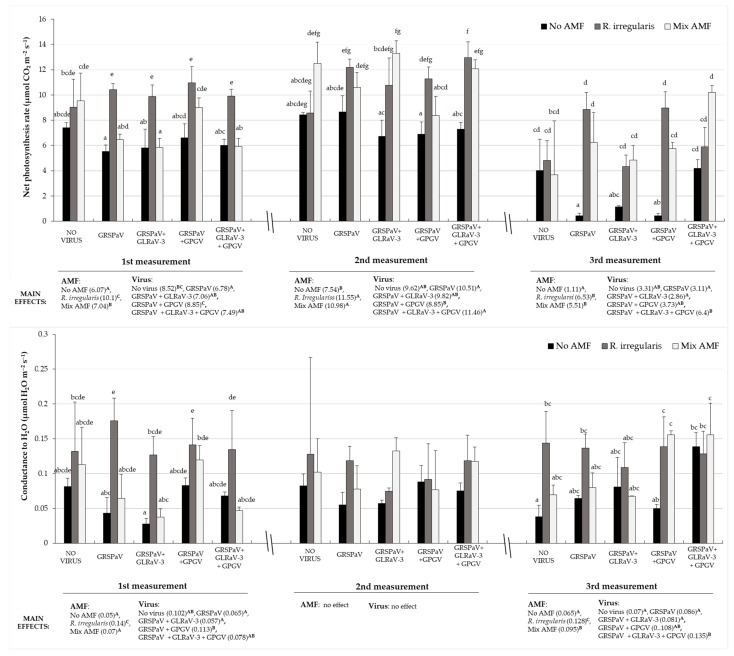
Effects of AMF inoculation on net photosynthesis rate (**top**) and conductance to H_2_O (**bottom**) shown in three measuring points during the growing season of grapevine infected by different combinations of GRSPaV, GLRaV3 and GPGV viruses. Measuring was done in May (1st), June (2nd) and September (3rd). Two-way ANOVA was made for each measurement with uppercase letters indicating statistically significant difference in main effects with means in brackets. Treatments with distinct lowercase letters indicate a statistically significant difference in each measurement (*p* < 0.05) determined by the Bonferroni post-hoc test.

By comparing treatments with viruses only (T4, T7, T10 and T13), we could estimate if virus combinations caused changes in grapevine’s measured parameters compared to control (T1) and how it relates to the treatment inoculated with AMF (Figure 1). In first and second measuring point there was no difference in net photosynthesis rate of virus infected plants (T4, T7, T10 and T13) compared to virus free control (T1). However, decreased values of net photosynthesis rate were observed at third measurement where GRSPaV (T4) and GRSPaV + GPGV (T10) were present, while GRSPaV + GLRaV-3 (T8, T9) treated plants had lower, but insufficiently significant, net photosynthesis rate. For the conductance to H_2_O, only GRSPaV + GLRaV-3 + GPGV (T13) infected plants had significantly higher values than virus free control, evident only at the third measurement. GRSPaV + GLRaV-3 infected plants along with GRSPaV infected plants expressed faster response to virus infection (first measuring point), through reduced transpiration rate and intercellular CO_2_ concentration compared to virus-free control.

These observations were used to estimate whether AMF addition would change virus effect by performing two-way ANOVA. For the net photosynthesis rate, the positive effect of AMF was the most obvious out of all gas exchange parameters (Figure 1). At all three measuring points, this photosynthetic parameter was significantly higher in treatments were *R. irregularis* (T2, T5, T8, T11 and T14) or Mix AMF (T3, T6, T9, T12 and T15) were added, compared to the treatment where only viruses were present. During the first measurement net photosynthesis rate was significantly enhanced, mostly in *R. irregularis* inoculated, virus infected grapevine plants (T5, -8, -11, -14). During the following months, Mix AMF also caused significant increase compared to non-AMF controls, especially in the second measuring point for treatments involving GLRaV-3 (T9, T15). In the final measuring point, GRSPaV and GRSPaV + GPGV infected plants, had the most significant induction of net photosynthesis rate regardless of AMF inoculum used. Repeated measures ANOVA revealed that there were significant changes between the measurements in observed parameters during the studied period, particularly for net photosynthetic activity, which decreased from first and second to the third measurement in all treatments (*p* < 0.001). Conductance to H_2_O was also significantly influenced by the added AMF, especially at the first measurement (May), at the point of early virus and AMF infection, where GRSPaV (T4) and GRSPaV + GPGV (T10) treated plants were most responsive to *R. irregularis* inoculation (Figure 1). Also, for transpiration and intercellular CO_2_ concentration two-way ANOVA revealed significant interaction between two independent factors: virus and AMF status. Regarding transpiration, this interaction (F = 3.150, *p* = 0.01) pointed out that this parameter was significantly higher in GRSPaV and GRSPaV + GLRaV3 treatments when *R. irregularis* was inoculated, compared to treatments where mix AMF inoculum was added or to treatments without AMF. For intercellular CO_2_ concentration, although significant interaction (F = 4.24, *p* = 0.02) was found, only one treatment stands out (GRSPaV + GLRaV3, without AMF) being lower from all the others.

**Figure 2:**
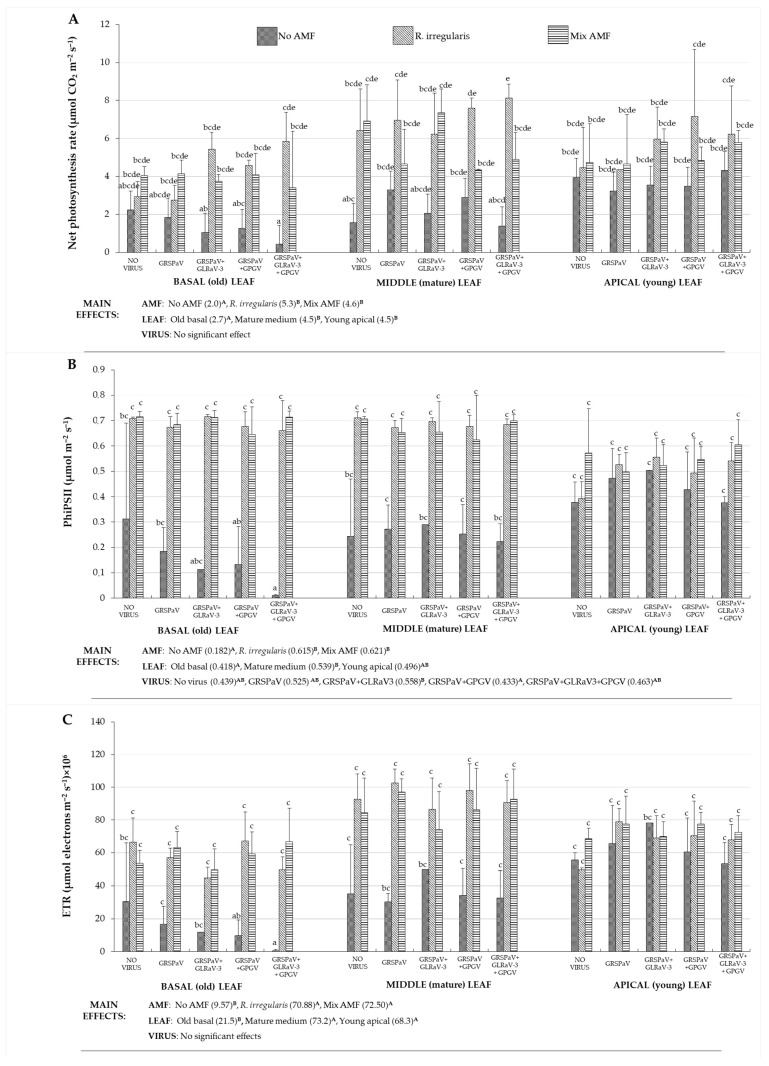
Effects of AMF inoculation on net photosynthesis rate (**A**), quantum efficiency in light (**B**), and electron transport rate (**C**) in the grapevine leaves of different maturity infected with GRSPaV, GLRaV3 and/or GPGV viruses. Parameters were analyzed by three-way ANOVA and statistically significant differences in main effects are indicated by distinct uppercase letters. Distinct lowercase letters represent statistically significant difference (*p* < 0.05), made with Bonferroni post-hoc test.

Three months after AMF inoculation additional measurements of photosynthetic parameters were performed on three leaves per plant: old-basal leaf, mature-medium leaf and young-apical leaf. Three-way ANOVA revealed no interaction virus × AMF × leaf type (F = 1.764, *p* = ns) for the net photosynthesis rate where this parameter was related to the leaf type (F = 22.367, *p* < 0.001) and AMF status of the treatment (F = 63.586, *p* < 0.001) but not to the type of virus combination (non-significant; Figure 2). On the other hand, for the quantum efficiency in light (Φ_PSII_) and electron transport rate (ETR) significant interactions virus × AMF × leaf type was found (F = 1.828, *p* = 0.035 and F = 1.93, *p* = 0.023, respectively). For both of these parameters AMF was the factor that influenced them the most (F = 76.78, *p* < 0.001 and F = 13.61, *p* < 0.001 respectively), followed by the type of the leaf (F = 11.93, *p* < 0.001 and F = 12.91, *p* < 0.001 respectively). For all three parameters in Figure 2, the lowest values were measured in old basal leaf. No significant differences were found between two types of AMF inoculum, but both were generally represented with values higher from the non-AMF controls. Although independent factor of virus status gave no significant effects in three-way ANOVA, significantly increased parameters’ values in mycorrhized vs. non-mycorrhized treatments were found in treatments GRSPaV + GPGV and GRSPaV + GLRaV3 + GPGV.

### 2.3. Pigment Concentrations

“NO AMF” treatments (T4, -7, -10, -13), containing only viruses, showed no significant difference compared to the healthy control. However, addition of AMF brought significant increase above their non-AMF control, particularly for the treatment GRSPaV + GLRaV-3 (Table 2). Contrarily to LiCor parameters, pigments concentrations revealed higher values when Mix AMF were in inoculum than when *R. irregularis* alone was added. Two-way ANOVA revealed significant interactions between AMF and virus compositions influencing chlorophyll a (F = 2.270, *p* = 0.045) and total chlorophyll (F = 2.263, *p* = 0.046). However, majority of pigment accumulation, mainly chlorophyll a and carotenoids, was significantly increased due to AMF inoculum, particularly in treatment with Mix AMF.

### 2.4. Plant Growth

Six months after virus inoculation, there was no significant influence of only viruses on grapevines, compared to virus-free control. Similarly, addition of AMF inoculum had no significant effect on plant growth. However, content of dry matter in total fresh weight was significantly influenced both by viruses and AMF inoculation (F = 2.73, *p* = 0.016). Regarding AMF inoculum, *R. irregularis* treated plants have higher dry mater content than Mix AMF treated plants, while treatments without AMF had the lowest dry matter content. For data on plant growth and tissue weight ratios refer to Table S1.

## 3. Discussion

In this paper, effects of AMF on grapevine photosynthesis in simultaneous coinfection with virus have been investigated. So far, the negative effects of grapevine viruses, particularly GLRaV-3 [3,10,11,34] and the positive effects of AMF on grapevine photosynthesis and photosynthesis-related parameters have been reported [35,36,37]. However, there is a gap in research of their interactive effects in perennial plants and up to now no investigation on virus—AMF interactions with grapevine physiology was reported.

During this study we hypothesized that AMF have the potential to modify effects of viruses of different pathogenicity on photosynthesis in grapevine hosts. To verify this hypothesis, we observed plants infected with only viruses and the corresponding treatments with added AMF. Regarding the former ones [15,17], the latest measurement revealed only significantly reduced net CO_2_ assimilation. Interestingly, in this case grapevine solely infected with GRSPaV had lower net photosynthesis rate than any other virus combination. Further, concentration of chlorophyll *a*, chlorophyll *b* and total carotenoids were not affected with GRSPaV, with no difference between treatments with or without AMF. This strong effect of GRSPaV on decreasing the net photosynthesis rate while having almost no effect on leaf chlorophyll content was already shown [15]. The underlying reason for that could be due to potential beneficial role of GRSPaV, that was proposed by some authors [15,16].

In accordance to described virus induced changes, further estimations were performed on the effects of AMF in selected treatments. This study proved that the presence of AMF associations greatly influenced grapevine response in parameters linked to photosynthesis. The net photosynthesis rate has been repeatedly higher in AMF inoculated plants compared to virus infected, AMF free plants. Furthermore, AMF inoculum composition seems to play an important role since single species AMF inoculum (*R. irregularis)* induced greater changes than inoculum composed of three species (*R. irregularis*, *F. mosseae*, *F. caledonium*). Similar results have been reported with grapevine facing water stress, where AMF contributed to greater photosynthetic rate, but also conductance to H_2_O and transpiration rate [22]. The discrepancies in first measurement of net photosynthesis rate between one-species and mix mycorrhizal inoculum may be due to possible competition interplay or simply prolonged phase of symbiosis establishment for mixed mycorrhizal inoculum as seen from significantly fewer arbuscular and vesicular structures present in the roots inoculated by mixture of AMF. There have been reports of different influence of single versus mixed AMF inoculum on plant growth and physiology in the context of functional complementarity or competition regarding relatedness of AMF species used [37,38] Different influence of single vs. mix AMF on plant physiology is still topic to be further elucidated. However, our results indicate that effects of *R. irregularis* and mix AMF species is primarily significant during first measurement and diminished over time. Although their total colonization rates were similar, higher arbuscular and even more vesicular abundances in *R. irregularis* treatments, found in our study, indicate the possibility of different rates of symbiotic association establishment.

Concurrent appearance of GRSPaV and AMF in the grapevine is present in vineyards worldwide, frequently coinfected with GLRaV-3 and GPGV. Hence, GRSPaV–AMF–grapevine interactions may be observed as a model multipartite biosystem for investigating different variations of virus–AMF relationship with the grapevine. It would be interesting to explore, on transcriptomic level, if the synergistic interplay between GRSPaV and a specific mycorrhizal specie exists that could be utilized in agricultural regions heavily infected with viruses.

In this study, the treatments containing GLRaV-3 had the most severe depletion of chlorophyll *a* and total carotenoid concentration, the observation that was reported in published literature and explained by heightened chlorophyllase activity [39]. However, the net photosynthesis rate did not reflect severe effect of GLRaV-3 coinfection more than with other viral treatment. The coinfection of GRSPaV with detrimental viruses such as GLRaV-3 or GPGV was intended to provoke more severe host reaction, but the response was similar across viral treatments. The reason for that could be a short infection period or no underlying interaction among viral species, as pointed out for closely and distantly related viruses [39,40,41]. Moreover, regarding pigment concentrations, grapevine colonized with mixed AMF performed better than those inoculated with single AMF, *R. irregularis*.

The analysis of different leaf age regarding photosynthetic parameters revealed that basal, oldest leaves had most perturbed net photosynthesis rate. This observation is in contrast to field grown grapevine where basal leaves maintain photosynthetic ability over long period of time [42]. This trend is connected to the favorable conditions, whereas in grapevine challenged with virus induced stress, photosynthetic perturbances could occur more easily in older leaves than the younger ones since the accumulation of viral titer is expectantly highest in older leaves [43], which is confirmed by our results. AMF caused increased net photosynthesis rate and electron transport rate, again the least intensively in oldest leaves. Maximum photosynthetic performance of the leaves is found to be reached with the onset of chlorophyll content decrease [44]. Since AMF inoculum has an impact on pigment concentration, the delayed response and discordance of net photosynthesis rate between treatments could result in basal leaves maintaining photosynthetic activity longer into the growing season than the basal leaves of AMF free grapevines. Even though viral induced stress did not significantly disturb quantum efficiency in light or electron transport rate, those two parameters were significantly upregulated in the presence of mycorrhizal fungi.

In summary, this study presents first insight into the complex interplay between viruses, AMF and grapevine as a host. The results contribute to the efforts to elucidate complex and underexplored niche of AMF mediated plant response to viral induced stress. Viral influence on grapevine photosynthesis and photosynthesis related parameters is shown to be mitigated by AMF colonization. Different levels of viral stress inducers through the use of selected viral infections, only partially produced differential effect on grapevine photosynthesis and photosynthesis related parameters, possibly due to short period of vine exposure to viruses. However, the addition of arbuscular mycorrhizal fungi, especially of mono species inoculum (*R. irregularis)*, resulted in induction of net photosynthesis rate, transpiration, conductance to H_2_O, quantum efficiency in light and electron transport rate, as well as increased chlorophyll and carotenoids concentrations and dry matter content in some cases. The beneficial role of AMF was especially seen in cases when only GRSPaV was present as a source of stress and in cases of GRSPaV coinfection with GLRaV-3 or GPGV. In virus infected grapevine mixed AMF inoculum reduced loss of leaf pigments more than *R. irregularis* alone. The presented results indicate that arbuscular mycorrhizal fungi can be beneficial for grapevine facing viral infection, in the context of functional physiology and cause enhanced photosynthesis, which is the basis for its growth and development.

## 4. Materials and Methods

### 4.1. Experimental Setup

**Figure 3:**
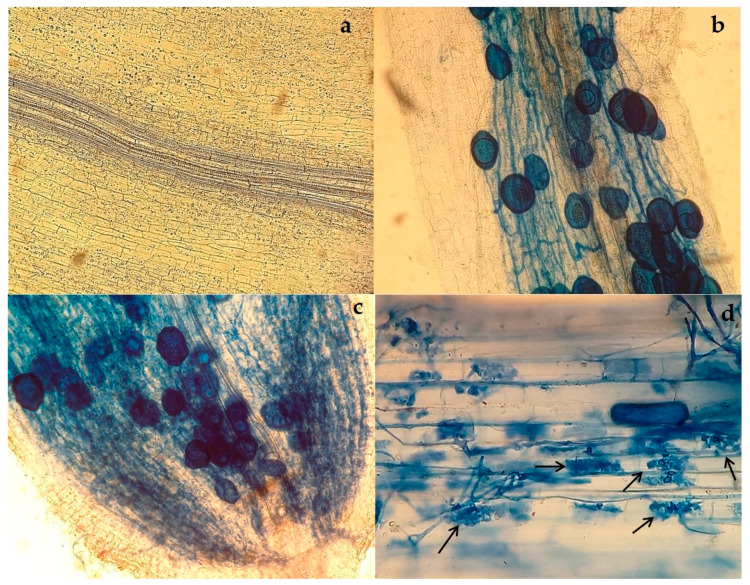
Microscopic view (×200) of grapevine roots treated with Trypan dye. Photos are representative of three different inoculums. Treatments 1, 4, 7, 10 and 13 are inoculated with unviable AMF inoculum (**a**), treatments 2, 5, 8, 11 and 14 are inoculated with *R. irregularis* (**b**) and treatments 3, 6, 9, 12 and 15 are inoculated with ‘MIX AMF’ consisting of *R. irregularis*, *F. mosseae* and *F. caledonium* (**c**). Arbuscules are indicated with arrows (**d**).

The Kober 5BB rootstock (*Vitis berlandieri* Planch. × *Vitis riparia* Michx.) was grafted with Merlot (*Vitis vinifera* L.) scions (both of Vitipep’s, Sarrians, France) and rooted in 6L pots in the greenhouse. Substrate mixture was autoclaved two times at 121 °C for 30 min prior to transplanting. Mixture consisted of soil, perlite, peat and quartz sand in 1:1:1:1/3 ratios, respectively. For the successfully developed plants, leaves were sampled for RNA isolation and detection of GLRaV-1, -2, -3, GVA, GVB, GFkV, GFLV, ArMV, GRSPaV [45], and GPGV [46]. The uninfected grapevines and those which harbored only GRSPaV were used in further steps. Plants that tested positive for any of the other viruses were excluded from the subsequent experimental setup. The two grapevine groups (‘GRSPaV positive’ and ‘no virus’) were infected with desired viruses through “chip budding” method with buds of known viral status in early February. Each plant received two buds from grapevine originating from collection vineyard (Institute of Adriatic Crops and Karst Reclamation). The buds were used as a source of GLRaV-3, GPGV or had no viruses. First indication of successful viral transmission by chip budding came after the grafted buds started growing [47]. To confirm the successful transmission of viruses from infected buds into the grapevine plant, virus detection of GLRaV-3 and GPGV was carried out as explained in the section ‘virus detection’. Up to that juncture, five grapevine groups were formed based on their virus status. Each group was subsequently treated with three mycorrhizal inoculums. Inoculation was carried out using one AMF species *Rhizophagus irregularis* (Symplanta LLC, Darmstadt, GE), mixture of *Rhizophagus irregularis*, *Funneliformis mosseae* and *Funneliformis caledonium* (Inoq LLC, Schnega, Germany) or autoclaved inactive AMF inoculum for mock inoculation. In described way 15 treatments were created in total (Table 1). Two months later (late March) mycorrhizal presence was checked to confirm successful colonization of AMF inoculated plants and lack of AMF presence in mock inoculated plants (Figure 3). The AMF detection was done in order to set up the treatments for analyzing the interactive effects of AMF and viruses on grapevine photosynthesis-related parameters. The final treatments were distributed inside a greenhouse using randomized complete block design and each treatment was composed of six biological replicates. Plants were watered regularly, and nutrition was supplemented every 3 or 4 weeks during the duration of the experiment with half strength Hoagland solution [48]. Regular procedures of grapevine protection against pests and diseases were performed as needed, without using copper-based fungicides for the leaves [49]. Three biological replicates per treatment were measured for analysis of the selected gas exchange, plant growth and pigment concentration variables.

### 4.2. Virus Detection

For virus detection, 100 mg of leaf tissue per sample was used to extract total RNA [45]. The quality and amount of RNA was assessed with Nanodrop^TM^ One spectrophotometer (Thermo Fisher Scientific, Waltham, MA, USA) by determining the spectrophotometric absorbance and ratios of A_260_/A_230_ and A_260_/A_280_. Complementary DNA was synthesized using M-MLV Reverse Transcriptase (Thermo Fisher Scientific, USA) following manufacturers guidelines. Detection of GRSPaV, GLRaV-3 and GPGV was done by using one technical replicate of each sample and amplifying using iTaq Universal SYBR Green Supermix (Bio-Rad, Hercules, CA, USA), 0.25 µM of each primer (Table 3), and cDNA sample diluted 1:10. Cycling conditions consisted of initial denaturation at 95 °C for 10 min, followed by 40 cycles at 95 °C/15 s, and 60 °C/1 min (CFX96 Touch Real-Time PCR, Bio-Rad, USA). The samples with Ct < 35 and with proper melting temperature data were considered positive. The final detection resulted in treatments setup as described in the Table 1.

### 4.3. Mycorrhizal Root Colonization Assessment

The detection of mycorrhizal association present in the roots was done two months after the inoculation, using Trypan blue as a coloring agent [52]. Fine grapevine roots were sampled and rinsed in water, cut to 1 cm segments and autoclaved at 121 °C for 5 min in 10% KOH. Subsequently, the roots were rinsed in distilled water and left for 5 min in 1% HCl. After that, roots were rinsed and stained with Trypan blue overnight. Finally, roots were rinsed, kept in 50% glycerol and 20 segments were mounted on slide. Under a compound microscope the total root colonization was estimated by examination of ~150 fields including assessment of arbuscules, vesicles and only hyphae according to the magnified intersections method [53]. Roots without cortex were excluded from the assessment. 

### 4.4. Gas Exchange

Gas exchange was measured on upper fully developed leaf between 09:00 a.m. and 11:00 a.m. in vivo, using non-destructive method with an open gas exchange system (Li-6400; Li-Cor. Inc., Lincoln, NE, USA). The variables measured were net photosynthesis rate (A_N_), leaf transpiration (E), intercellular CO_2_ concentration (C_i_) and conductance to H_2_O (g_s_). The measurements were performed with device parameters as follows: CO_2_ leaf chamber concentration was set at 400 ppm, saturated red light (500 µmol m^−2^ s^−1^) with addition of 10% blue light, relative air humidity of 50% and block temperature of 30 °C. Photosynthetic parameters were measured three times after the final inoculation with AMF (PI—post inoculation), as follows: two-, three- and five-months post inoculation, 2PI, 3PI, 5PI, respectively. Additionally, quantum efficiency in light (Φ_PSII_) and electron transport rate (ETR) were measured using compact porometer with pulse-amplitude modulation fluorometer Li-600 Porometer/Fluorometer (Li-Cor. Inc., Lincoln, NE, USA). Light-adapted leaf measurement was chosen, with auto gsw+F configuration. After enabling stability of the instrument, plants were surveyed under ambient conditions. Measurement of Φ_PSII_, ETR and gas exchange parameters were done three months post inoculation (3PI) for three leaves per plant differing in age and developmental phase. The measurements were made for the basal leaf (from the lower part of the plant), upper fully developed leaf (middle part of the plant) and apical-not fully developed leaf (upper part of the plant).

### 4.5. Pigment Analysis

Pigment concentrations were measured once, at 3PI, using fully developed leaves from three biological replicate per each treatment. The powder of freeze-dried fully-grown grapevine leaves was used for pigment analysis. Pigments were extracted from 10 mg of the plant material with 95% ethanol (overnight at room temperature in dark). Absorbances were measured spectrophotometrically at 470 nm, 647 nm and 663 nm. Chlorophyll *a*, chlorophyll *b* and total carotenoids were quantified using empirical equations, as well as chlorophyll a/chlorophyll b and total chlorophyll/total carotenoids ratios [54].

### 4.6. Grapevine Growth Parameters

At 3PI, shoot length and number of internodes of the grapevine plants were measured. The mean internode length was calculated by dividing total shoot length with number of internodes. Prior to pigment analysis, fresh and dry leaf weight were measured in order to calculate dry matter content in total weight. Leaves were freeze-vacuum dried at −50 °C, under 200 mbar vacuum.

### 4.7. Statistical Analysis

For statistical analysis two-way and three-way ANOVA as well as repeated measures ANOVA were performed in the Statistica 14.0.1. software (Tibco, Arlington, VA, USA), using Bonferroni post-hoc test (*p* < 0.05). Prior to statistical analysis data was transformed using natural logarithm in order to follow normal distribution.

## Figures and Tables

**Table 1:** Basic description and root AMF colonization percentages of the treatments used in the research. The colonization is shown as an average percentage ± standard deviation.

Treatment	Type of Inoculum (Factor)		Colonisation Percentage			
	Virus Status	Mycorrhizal Status (AMF)	Arbuscules (%)	Vesicles (%)	Hyphae Only (%)	Total%
T1	No virus	No AMF	Ø ^a^	Ø ^a^	Ø ^a^	Ø ^a^
T2		*Rhizophagus irregularis*	66.1 ± 13.2 ^b^	44.3 ± 24.1 ^bcd^	12.5 ± 4.8 ^bc^	78.6 ± 8.4 ^b^
T3		Mix *	75.6 ± 15.6 ^b^	14.8 ± 6.1 ^bcd^	15.8 ± 9.9 ^bc^	92.4 ± 4.7 ^b^
T4	GRSPaV	No AMF	Ø ^a^	Ø ^a^	Ø ^a^	Ø ^a^
T5		*Rhizophagus irregularis*	88.7 ± 12.4 ^b^	76.8 ± 19.9 ^cd^	5.7 ± 6.2 ^abc^	94.3 ± 6.2 ^b^
T6		Mix *	55.1 ± 10.8 ^b^	9.7 ± 4.9 ^b^	26 ± 7.4 ^c^	81.4 ± 9 ^b^
T7	GRSPaV + GLRaV-3	No AMF	Ø ^a^	Ø ^a^	Ø ^a^	Ø ^a^
T8		*Rhizophagus irregularis*	93.7 ± 4.7 ^b^	82.4 ± 9.7 ^cd^	3.1 ± 3.1 ^abc^	97.5 ± 1.1 ^b^
T9		Mix *	68.8 ± 17.5 ^b^	18.2 ± 7.1 ^bc^	18.2 ± 9.5 ^bc^	87.6 ± 10.7 ^b^
T10	GRSPaV + GPGV	No AMF	Ø ^a^	Ø ^a^	Ø ^a^	Ø ^a^
T11		*Rhizophagus irregularis*	86.8 ± 10.1 ^b^	65.4 ± 15.7 ^cd^	3.9 ± 3.7 ^ab^	90.6 ± 8.2 ^b^
T12		Mix *	85 ± 7.9 ^b^	28.3 ± 12.1 ^bcd^	10.7 ± 6.4 ^bc^	96 ± 2.7 ^b^
T13	GRSPaV + GLRaV-3 + GPGV	No AMF	Ø ^a^	Ø ^a^	Ø ^a^	Ø ^a^
T14		*Rhizophagus irregularis*	86.1 ± 11.8 ^b^	71.5 ± 11.7 d	5.8 ± 3.7 ^bc^	94.3 ± 5 ^b^
T15		Mix *	85.8 ± 8.6 ^b^	34.1 ± 14.4 ^bcd^	7.4 ± 1.9 ^bc^	93.4 ± 8.2 ^b^
Main Effects	Virus	No virus	47.5 ± 38.6	20.0 ± 25.3	5.0 ± 4.2	18.6 ± 10.5
		GRSPaV	60.0 ± 30.3	31.3 ± 32.0	8.3 ± 3.8	44.8 ± 5.3
		GRSPaV + GLRaV-3	61.2 ± 41.7	38.0 ± 38.3	3.6 ± 4.2	29.0 ± 8.5
		GRSPaV + GPGV	70.4 ± 33.3	40.3 ± 30.8	3.8 ± 3.7	44.9 ± 5.7
		GRSPaV + GLRaV-3 + GPGV	70.9 ± 32.3	46.1 ± 31.4	4.7 ± 2.5	44.7 ± 5.1
		*p*	ns	ns	ns	ns
	AMF	No AMF	0 ^a^	0 ^a^	0 ^a^	0 ^a^
		*Rhizophagus irregularis*	85.5 ± 13.0 ^c^	69.1 ± 19.9 ^c^	5.9 ± 4.9 ^b^	92.1 ± 8.0 ^c^
		Mix *	70.1 ± 18.0 ^b^	20.9 ± 13.8 ^b^	16.7 ± 10.1 ^c^	87.2 ± 11.0 ^b^
		*p*	<0.001	<0.001	<0.001	<0.001
	Virus × AMF	F	0.732	0.486	0.776	1.11
		*p*	ns	ns	ns	ns

* *Rhizophagus irregularis*, *Funneliformis mosseae* and *Funneliformis caledonium*; GRSPaV—grapevine rupestris stem pitting virus, GLRaV-3—grapevine leafroll associated virus 3, GPGV—grapevine pinot gris virus; lowercase letters indicate significant difference based on two-way ANOVA (*p* < 0.05).

**Table 2:** Measurement of leaf chlorophyll a and b, total leaf chlorophyll and carotenoids concentration, as well as ratios of chlorophyll a and b, and total chlorophyll and carotenoids of grapevine.

Treatment	Virus StaTUS	AMF Status	Chlorophyll ^a^	Chlorophyll ^b^	Total Chlorophyll	Total Carotenoids	Chlorophyll ^a^/ Chlorophyll ^b^	Chlorophyll/ Carotenoids
T1	NO VIRUS	NO AMF	1.43 ± 0.10 ^ab^	0.85 ± 0.10	2.28 ± 0.19 ^ab^	0.52 ± 0.07 ^abc^	1.68 ± 0.08	4.39 ± 0.19
T2		*R. irregularis*	1.76 ± 0.40 ^ab^	0.56 ± 0.32	2.32 ± 0.72 ^ab^	0.76 ± 0.10 ^abc^	4.09 ± 1.62	2.97 ± 0.55
T3		MIX AMF	1.96 ± 0.43 ^b^	1.04 ± 0.16	3.00 ± 0.59 ^ab^	0.73 ± 0.24 ^abc^	1.87 ± 0.12	4.29 ± 0.59
T4	GRSPaV	NO AMF	1.65 ± 0.38 ^ab^	1.22 ± 0.45	2.87 ± 0.83 ^ab^	0.54 ± 0.01 ^abc^	1.44 ± 0.22	5.32 ± 1.44
T5		*R. irregularis*	1.59 ± 0.31 ^b^	1.28 ± 0.64	2.87 ± 0.91 ^b^	0.43 ± 0.14 ^abc^	1.59 ± 0.77	8.34 ± 5.29
T6		MIX AMF	2.15 ± 0.47 ^b^	1.21 ± 0.47	3.35 ± 0.89 ^b^	0.79 ± 0.15 ^bc^	1.94 ± 0.50	4.28 ± 1.10
T7	GRSPaV + GLRaV-3	NO AMF	0.71 ± 0.09 ^a^	0.36 ± 0.05	1.07 ± 0.14 **^a^**	0.37 ± 0.08 ^abc^	1.96 ± 0.04	2.93 ± 0.24
T8		*R. irregularis*	1.88 ± 0.27 ^b^	1.34 ± 0.61	3.22 ± 0.84 ^b^	0.54 ± 0.14 ^abc^	1.69 ± 0.73	6.49 ± 2.63
T9		MIX AMF	2.65 ± 0.37 ^b^	2.06 ± 0.16	4.71 ± 0.21 ^b^	0.70 ± 0.29 ^abc^	1.31 ± 0.29	8.01 ± 3.01
T10	GRSPaV + GPGV	NO AMF	1.40 ± 0.11 ^ab^	1.00 ± 0.31	2.40 ± 0.42 ^ab^	0.45 ± 0.09 ^abc^	1.50 ± 0.36	5.67 ± 2.02
T11		*R. irregularis*	1.88 ± 0.35 ^b^	1.04 ± 0.34	2.92 ± 0.60 ^b^	0.65 ± 0.19 ^abc^	1.91 ± 0.43	4.85 ± 1.83
T12		MIX AMF	2.24 ± 0.45 ^b^	1.11 ± 0.29	3.35 ± 0.65 ^b^	0.82 ± 0.21 ^bc^	2.11 ± 0.49	4.29 ± 1.08
T13	GRSPaV + GLRaV-3 + GPGV	NO AMF	1.37 ± 0.10 ^ab^	1.23 ± 0.27	2.61 ± 0.37 ^ab^	0.28 ± 0.08 ^a^	1.15 ± 0.16	9.52 ± 1.28
T14		*R. irregularis*	1.58 ± 0.11 ^ab^	0.96 ± 0.13	2.53 ± 0.23 ^ab^	0.57 ± 0.06 ^abc^	1.67 ± 0.17	4.52 ± 0.60
T15		MIX AMF	2.32 ± 0.61 ^b^	1.29 ± 0.59	3.61 ± 1.18 ^b^	0.82 ± 0.15 ^c^	1.92 ± 0.31	4.39 ± 1.17
Main Effects	Virus	No virus	1.7 ± 0.4	0.8 ± 0.3	2.5 ± 0.7	0.7 ± 0.2	2.5 ± 1.6	3.9 ± 0.9
		GRSPaV	1.8 ± 0.5	1.2 ± 0.6	3.1 ± 1.0	0.6 ± 0.2	1.7 ± 0.6	5.9 ± 3.9
		GRSPaV + GLRaV-3	1.6 ± 0.9	1.3 ± 0.8	3.0 ± 1.6	0.5 ± 0.2	1.7 ± 0.6	5.9 ± 3.3
		GRSPaV + GPGV	1.8 ± 0.5	1.1 ± 0.3	2.9 ± 0.7	0.7 ± 0.2	1.9 ± 0.5	4.8 ± 1.8
		GRSPaV + GLRaV-3 + GPGV	1.8 ± 0.6	1.2 ± 0.5	3.1 ± 1.1	0.7 ± 0.2	1.7 ± 0.4	5.2 ± 2.2
		*p*	ns	ns	ns	ns	ns	ns
	AMF	No AMF	1.3 ± 0.4 ^a^	0.9 ± 0.4	2.2 ± 0.8 ^a^	0.4 ± 0.1 ^a^	1.5 ± 0.3	5.6 ± 2.7
		*Rhizophagus irregularis*	1.7 ± 0.3 ^b^	1.1 ± 0.5	2.8 ± 0.8 ^ab^	0.6 ± 0.2 ^a^	2.0 ± 1.1	5.8 ± 3.7
		Mix *	2.2 ± 0.5 ^c^	1.3 ± 0.5	3.5 ± 1.0 ^b^	0.8 ± 0.2 ^b^	1.9 ± 0.5	4.7 ± 1.8
		*p*	<0.001	ns	<0.001	<0.001	ns	ns
	Virus × AMF	F	2.270	2.067	2.263	1.560	1.781	2.299
		*p*	0.045	ns	0.046	ns	ns	ns

* *Rhizophagus irregularis*, *Funneliformis mosseae* and *Funneliformis caledonium*; GRSPaV—grapevine rupestris stem pitting virus, GLRaV-3—grapevine leafroll associated virus 3, GPGV—grapevine pinot gris virus; Lowercase letters indicate the statistically significant difference revealed by two-way ANOVA (*p* < 0.05).

**Table 3:** Primers used for virus detection.

Target	Primer	Primer Sequences 5′–3′	Reference
GLRaV-3	Forward	TTGGTGGATGAGGTGCACAT	[50]
	Reverse	GTTGCGAAGACGCCTAGTTGT	
GRSPaV	Forward	GTGATCCATGTCAAAGCACATATG	[50]
	Reverse	CTCAGCGCCCAAAATTGC	
GPGV	Forward	GAATCGCTTGCTTTTTCATG	[51]
	Reverse	CTACATACTAAATGCACTCTCC	

## Data Availability

The data presented in this study are available on request from the corresponding author.

## Supplementary Materials

The following supporting information can be downloaded at: https://www.mdpi.com/article/10.3390/plants12091783/s1, Table S1: Growth parameters and dry content of grapevine interacting with AMF and viruses.
