# Supplementary material for: Arbuscular Mycorrhizal Fungi Induce Changes of Photosynthesis-Related Parameters in Virus Infected Grapevine
Source: Plants (Basel). 2023 Apr 26;12(9):1783. doi: 10.3390/plants12091783 (PMC10180532; doi:10.3390/plants12091783)
Supplement: Supplementary file 1 [file plants-12-01783-s001.zip › plants-2314918-supplementary.pdf]

Supplementary Table S1. Growth parameters and dry content of grapevine interacting with AMF and viruses. The data is presented as mean ± SD.

Treatment	Viral status	Shoot Length (cm)	Internode Length (cm)	DW/FW
T1	NO VIRUS	33.3 ± 5.30	1.6 ± 0.58	0.37 ± 0.01 [abcd]
T2		77.5 ± 12.02	2.5 ± 0.28	0.4 ± 0.02 [abcd]
T3		101.5 ± 19.09	2.9 ± 0.04	0.39 ± 0.01 [abcd]
T4	GRSPaV	51.0 ± 4.18	2.0 ± 0.22	0.36 ± 0.01 [abc]
T5		62.6 ± 3.07	2.5 ± 0.23	0.43 ± 0.01 [d]
T6		83.4 ± 16.74	2.5 ± 0.37	0.37 ± 0.02 [ab]
T7	GRSPaV + GLRaV-3	46.8 ± 6.72	2.0 ± 0.04	0.35 ± 0.01 [ab]
T8		62.3 ± 11.68	2.6 ± 0.43	0.41 ± 0.02 [bcd]
T9		71.3 ± 10.02	2.3 ± 0.06	0.39 ± 0.01 [abcd]
T10	GRSPaV + GPGV	48.7 ± 11.50	1.9 ± 0.35	0.4 ± 0.01 [abcd]
T11		64.8 ± 18.87	2.4 ± 0.28	0.4 ± 0.01 [abcd]
T12		78.0 ± 18.75	2.5 ± 0.38	0.4 ± 0.02 [abcd]
T13	GRSPaV + GLRaV-3 + GPGV	36.0 ± 2.83	1.7 ± 0.13	0.34 ± 0.01 [a]
T14		64.6 ± 13.91	2.5 ± 0.41	0.41 ± 0.02 [cd]
T15		59.0 ± 16.90	2.3 ± 0.66	0.38 ± 0.03 [abc]
Virus	No	70.8 ± 32.7	2.3 ± 0.6	0.38 ± 0.02
	GRSPaV	69.4 ± 18.3	2.4 ± 0.4	0.39 ± 0.03
	GRSPaV + GLRaV3	61.8 ± 13.3	2.3 ± 0.4	0.39 ± 0.03
	GRSPaV + GPGV	66.0 ± 13.8	2.3 ± 0.4	0.40 ± 0.02
	GRSPaV + GLRaV3 + GPGV	58.9 ± 19.4	2.3 ± 0.5	0.39 ± 0.04
	p	ns	ns	ns
AMF	No AMF	45.2 ± 9.1	1.9 ± 0.3	0.36 ± 0.02 [a]
	R. irregularis	65.0 ± 13.1	2.3 ± 0.3	0.41 ± 0.02 [c]
	Mix AMF	77.2 ± 21.3	2.5 ± 0.4	0.38 ± 0.02 [b]
	p	ns	ns	**< 0.001**
Virus × AMF	F	0.937	0.517	2.73
	p	ns	ns	**0.016**

The lowercase letters represent statistically significant difference in growth parameter revealed by two-way ANOVA.
